# Supplementary material for: EGCG Inhibits Tumor Growth in Melanoma by Targeting JAK-STAT Signaling and Its Downstream PD-L1/PD-L2-PD1 Axis in Tumors and Enhancing Cytotoxic T-Cell Responses
Source: Pharmaceuticals (Basel). 2021 Oct 26;14(11):1081. doi: 10.3390/ph14111081 (PMC8618268; doi:10.3390/ph14111081)
Supplement: Supplementary file 1 [file pharmaceuticals-14-01081-s001.zip › pharmaceuticals-1410480-supplementary.pptm]

## Slide 1
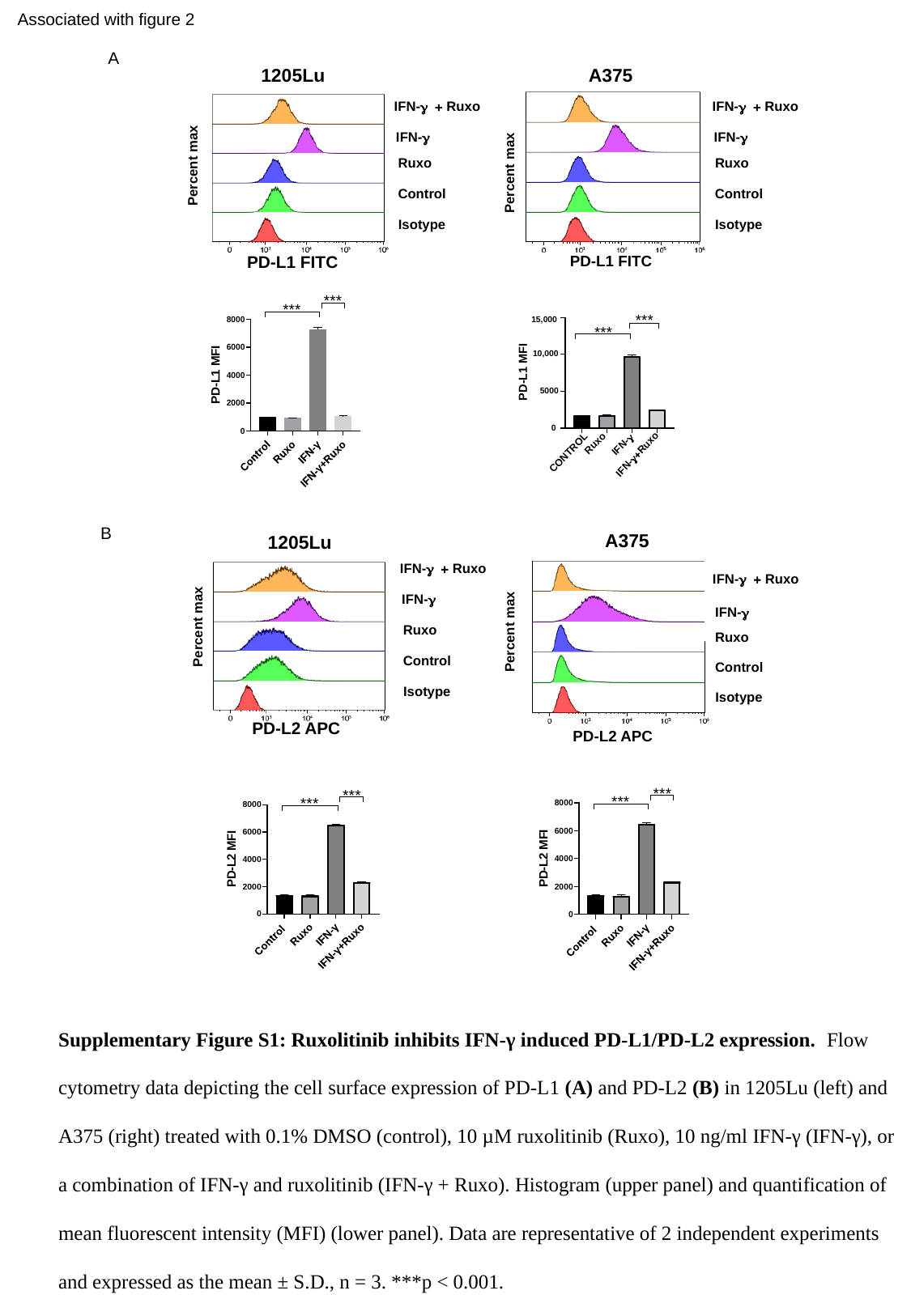

Associated with figure 2
A
1205Lu
A375
Ruxo
Control
Isotype
PD-L1 FITC
Percent max
IFN-g + Ruxo
IFN-g
IFN-g + Ruxo
IFN-g
Ruxo
Control
Isotype
Percent max
PD-L1 FITC
B
A375
1205Lu
IFN-g + Ruxo
Percent max
PD-L2 APC
Ruxo
Control
Isotype
IFN-g
Ruxo
Control
Isotype
PD-L2 APC
Percent max
IFN-g + Ruxo
IFN-g
15,000
10,000
5000
0
Ruxo
IFN-g
 CONTROL
IFN-g+Ruxo
PD-L1 MFI
Supplementary Figure S1: Ruxolitinib inhibits IFN-γ induced PD-L1/PD-L2 expression. Flow cytometry data depicting the cell surface expression of PD-L1 (A) and PD-L2 (B) in 1205Lu (left) and A375 (right) treated with 0.1% DMSO (control), 10 µM ruxolitinib (Ruxo), 10 ng/ml IFN-γ (IFN-γ), or a combination of IFN-γ and ruxolitinib (IFN-γ + Ruxo). Histogram (upper panel) and quantification of mean fluorescent intensity (MFI) (lower panel). Data are representative of 2 independent experiments and expressed as the mean ± S.D., n = 3. ***p < 0.001.

## Slide 2
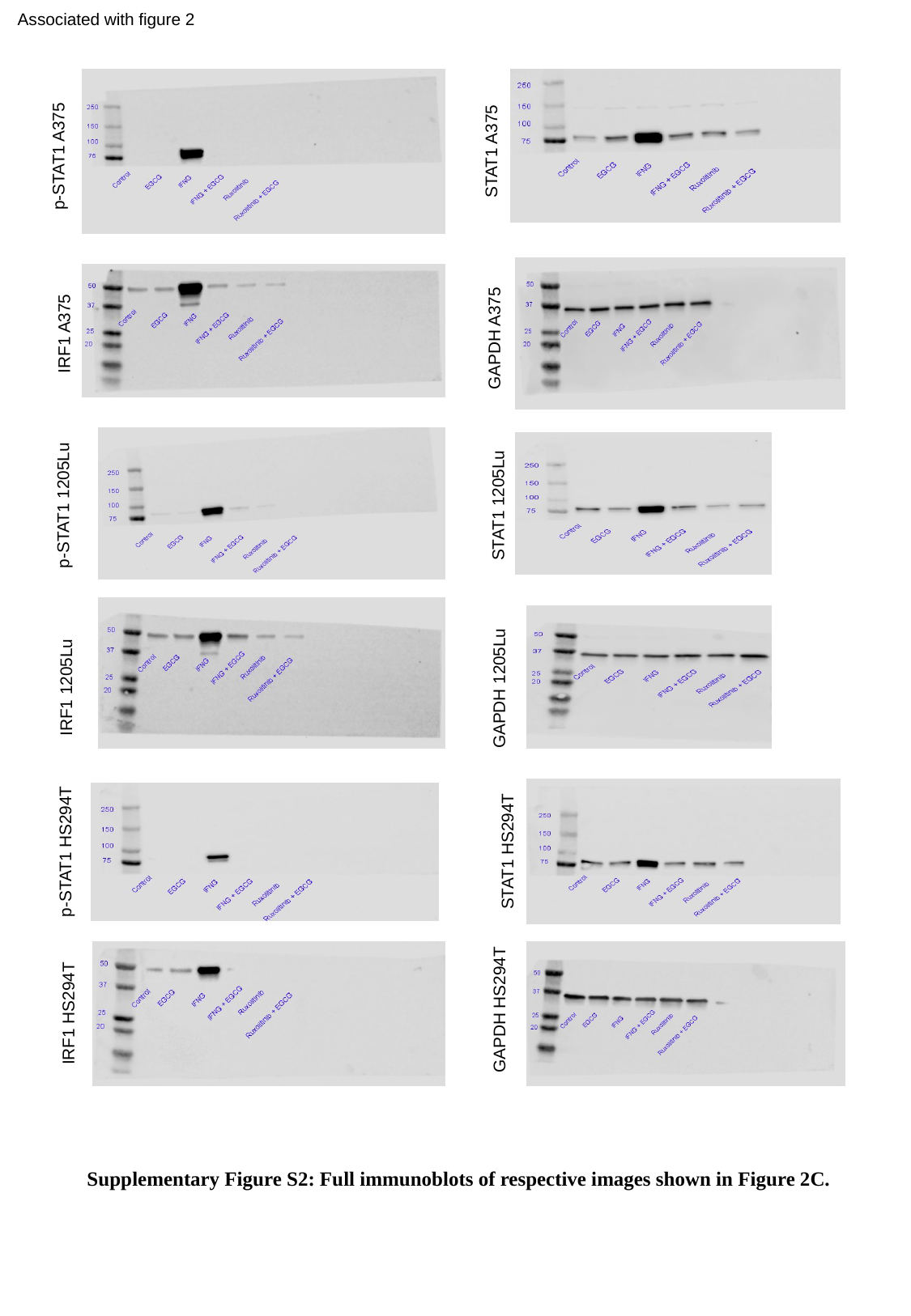

Associated with figure 2
p-STAT1 A375
STAT1 A375
IRF1 A375
GAPDH A375
p-STAT1 1205Lu
STAT1 1205Lu
IRF1 1205Lu
GAPDH 1205Lu
p-STAT1 HS294T
STAT1 HS294T
GAPDH HS294T
IRF1 HS294T
Supplementary Figure S2: Full immunoblots of respective images shown in Figure 2C.

## Slide 3
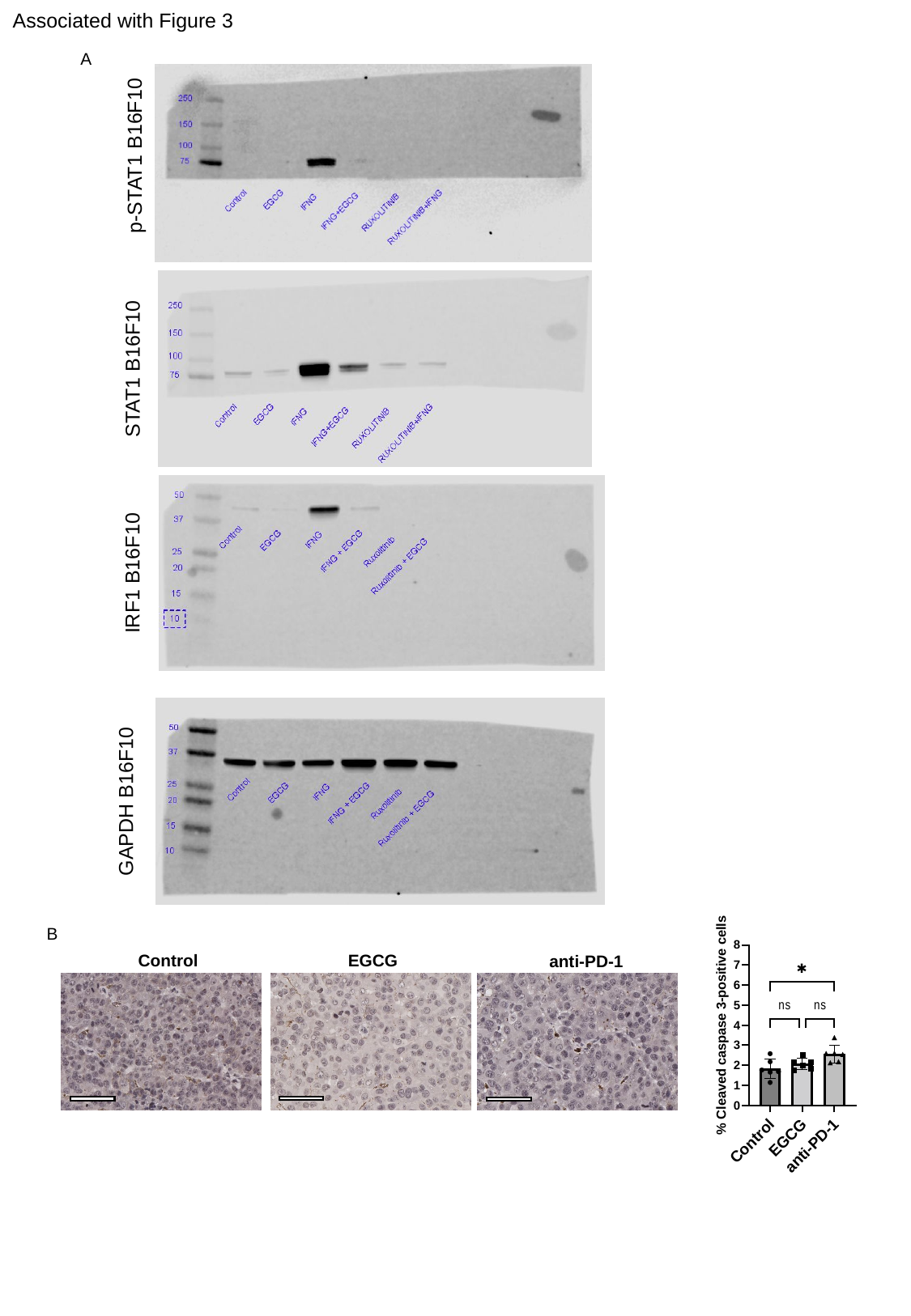

Associated with Figure 3
A
p-STAT1 B16F10
STAT1 B16F10
IRF1 B16F10
GAPDH B16F10
B
Control
EGCG
anti-PD-1

## Slide 4
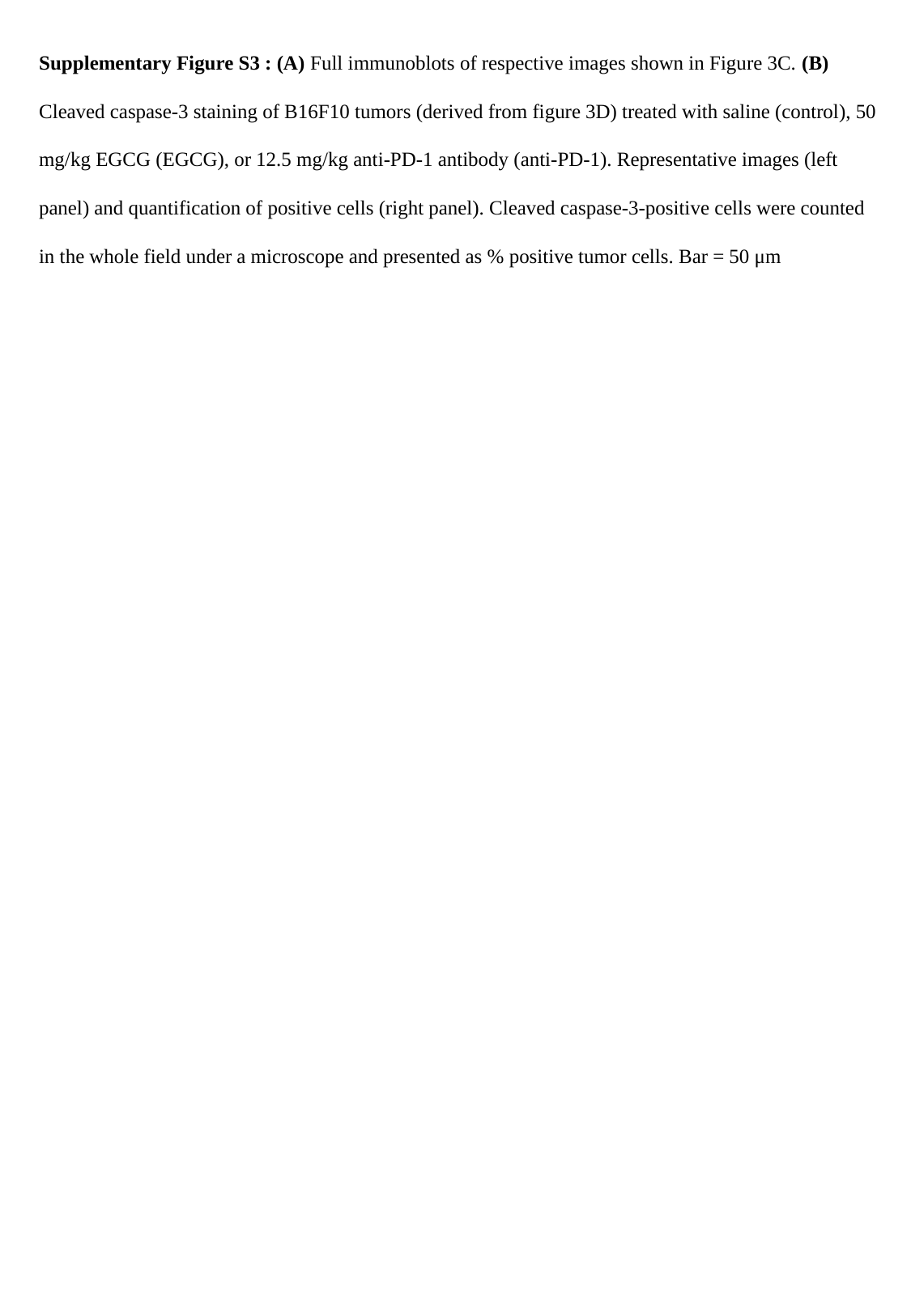

Supplementary Figure S3 : (A) Full immunoblots of respective images shown in Figure 3C. (B) Cleaved caspase-3 staining of B16F10 tumors (derived from figure 3D) treated with saline (control), 50 mg/kg EGCG (EGCG), or 12.5 mg/kg anti-PD-1 antibody (anti-PD-1). Representative images (left panel) and quantification of positive cells (right panel). Cleaved caspase-3-positive cells were counted in the whole field under a microscope and presented as % positive tumor cells. Bar = 50 μm

## Slide 5
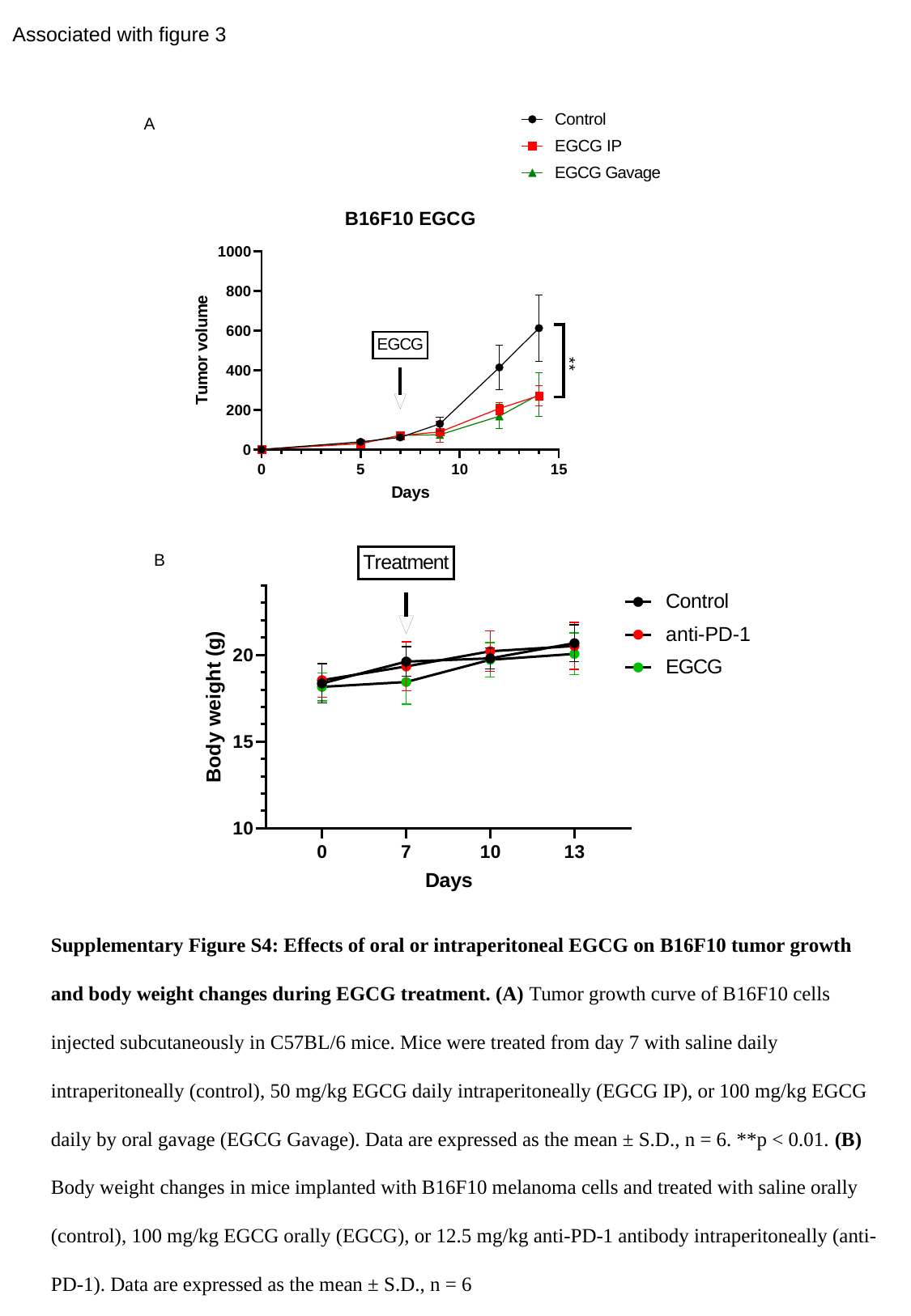

Associated with figure 3
A
B
Supplementary Figure S4: Effects of oral or intraperitoneal EGCG on B16F10 tumor growth and body weight changes during EGCG treatment. (A) Tumor growth curve of B16F10 cells injected subcutaneously in C57BL/6 mice. Mice were treated from day 7 with saline daily intraperitoneally (control), 50 mg/kg EGCG daily intraperitoneally (EGCG IP), or 100 mg/kg EGCG daily by oral gavage (EGCG Gavage). Data are expressed as the mean ± S.D., n = 6. **p < 0.01. (B) Body weight changes in mice implanted with B16F10 melanoma cells and treated with saline orally (control), 100 mg/kg EGCG orally (EGCG), or 12.5 mg/kg anti-PD-1 antibody intraperitoneally (anti-PD-1). Data are expressed as the mean ± S.D., n = 6

## Slide 6
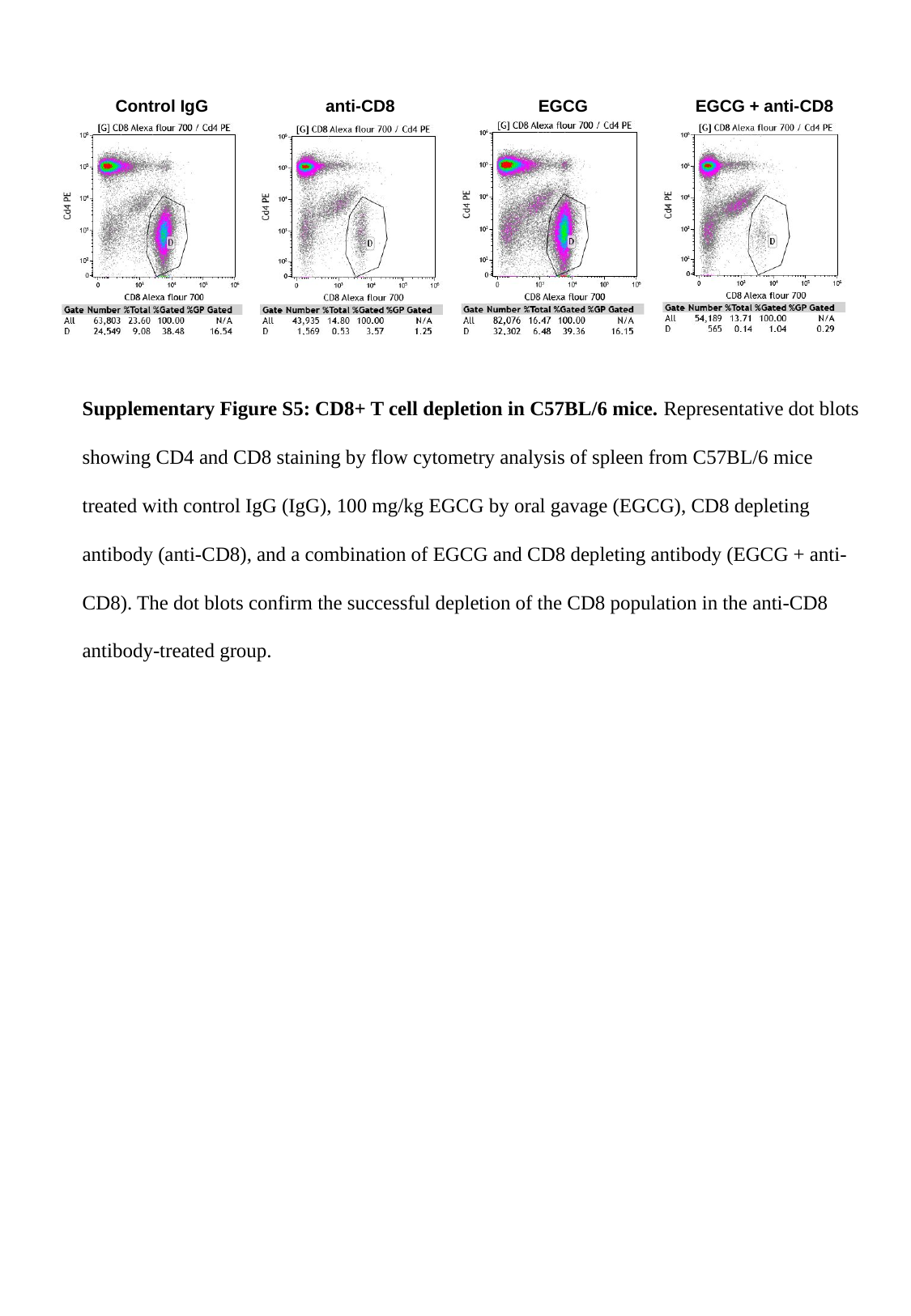

Control IgG
anti-CD8
EGCG
EGCG + anti-CD8
Supplementary Figure S5: CD8+ T cell depletion in C57BL/6 mice. Representative dot blots showing CD4 and CD8 staining by flow cytometry analysis of spleen from C57BL/6 mice treated with control IgG (IgG), 100 mg/kg EGCG by oral gavage (EGCG), CD8 depleting antibody (anti-CD8), and a combination of EGCG and CD8 depleting antibody (EGCG + anti-CD8). The dot blots confirm the successful depletion of the CD8 population in the anti-CD8 antibody-treated group.

## Slide 7
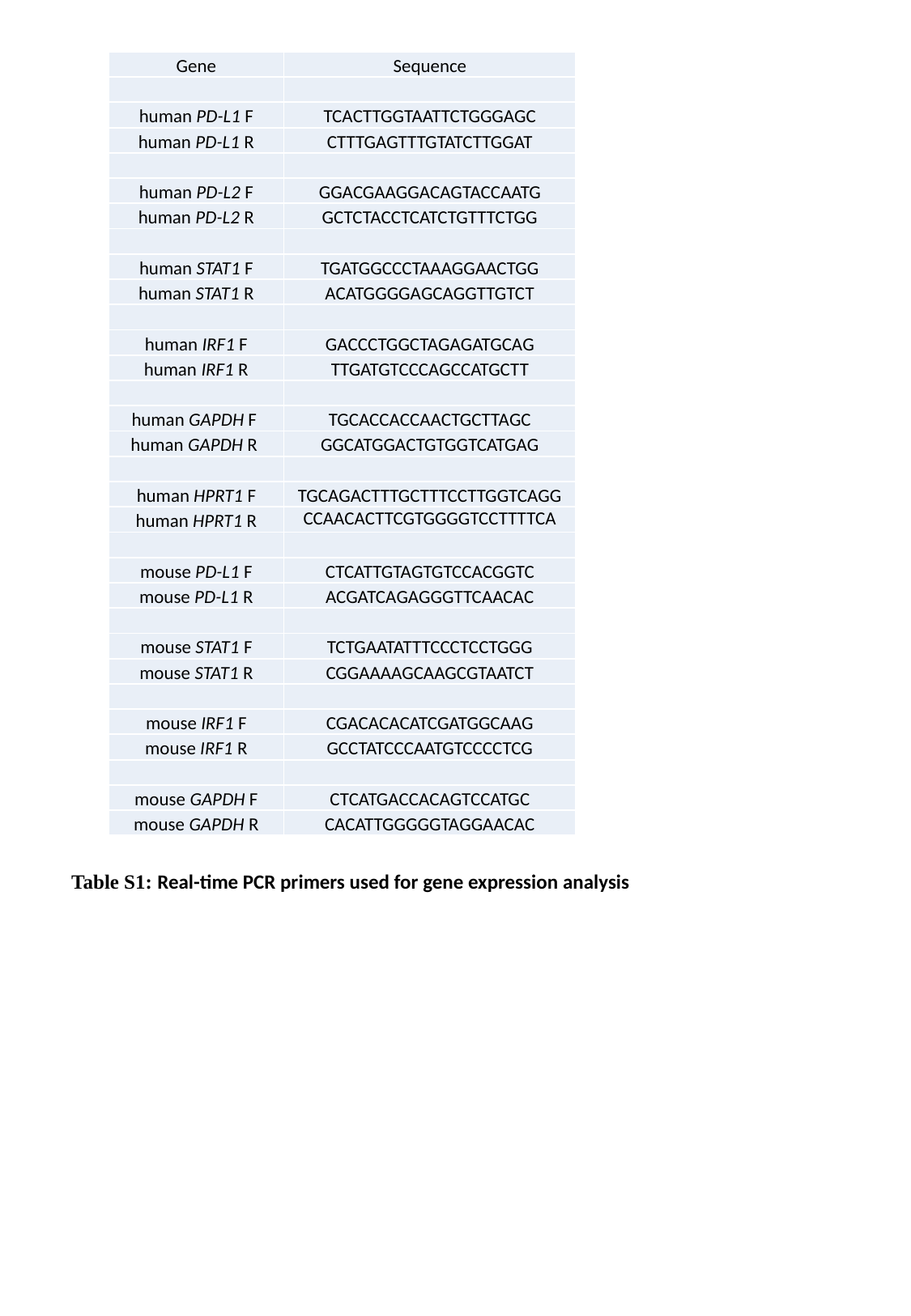

| Gene | Sequence |
| --- | --- |
| | |
| human PD-L1 F | TCACTTGGTAATTCTGGGAGC |
| human PD-L1 R | CTTTGAGTTTGTATCTTGGAT |
| | |
| human PD-L2 F | GGACGAAGGACAGTACCAATG |
| human PD-L2 R | GCTCTACCTCATCTGTTTCTGG |
| | |
| human STAT1 F | TGATGGCCCTAAAGGAACTGG |
| human STAT1 R | ACATGGGGAGCAGGTTGTCT |
| | |
| human IRF1 F | GACCCTGGCTAGAGATGCAG |
| human IRF1 R | TTGATGTCCCAGCCATGCTT |
| | |
| human GAPDH F | TGCACCACCAACTGCTTAGC |
| human GAPDH R | GGCATGGACTGTGGTCATGAG |
| | |
| human HPRT1 F | TGCAGACTTTGCTTTCCTTGGTCAGG |
| human HPRT1 R | CCAACACTTCGTGGGGTCCTTTTCA |
| | |
| mouse PD-L1 F | CTCATTGTAGTGTCCACGGTC |
| mouse PD-L1 R | ACGATCAGAGGGTTCAACAC |
| | |
| mouse STAT1 F | TCTGAATATTTCCCTCCTGGG |
| mouse STAT1 R | CGGAAAAGCAAGCGTAATCT |
| | |
| mouse IRF1 F | CGACACACATCGATGGCAAG |
| mouse IRF1 R | GCCTATCCCAATGTCCCCTCG |
| | |
| mouse GAPDH F | CTCATGACCACAGTCCATGC |
| mouse GAPDH R | CACATTGGGGGTAGGAACAC |
Table S1: Real-time PCR primers used for gene expression analysis
